# A State-of-the-Art Review on the Freeze–Thaw Resistance of Sustainable Geopolymer Gel Composites: Mechanisms, Determinants, and Models

**DOI:** 10.3390/gels11070537

**Published:** 2025-07-11

**Authors:** Peng Zhang, Baozhi Shi, Xiaobing Dai, Cancan Chen, Canhua Lai

**Affiliations:** 1 School of Water Conservancy and Transportation, Zhengzhou University, Zhengzhou 450001, China; zhangpeng@zzu.edu.cn (P.Z.); 15136736833@163.com (B.S.); daixb@zzu.edu.cn (X.D.); 13395998071@163.com (C.L.); 2 State Key Laboratory of Tunnel Boring Machine and Intelligent Operation, Zhengzhou 450001, China

**Keywords:** geopolymer gel composites, freeze–thaw resistance, deterioration mechanisms, influence factors, prediction models

## Abstract

Geopolymer, as a sustainable, low-carbon gel binder, is regarded as a potential alternative to cement. Freeze–thaw (F-T) resistance, which has a profound influence on the service life of structures, is a crucial indicator for assessing the durability of geopolymer composites (GCs). Consequently, comprehending the F-T resistance of GCs is of the utmost significance for their practical implementation. In this article, a comprehensive and in-depth review of the F-T resistance of GCs is conducted. This review systematically synthesizes several frequently employed theories regarding F-T damage, with the aim of elucidating the underlying mechanisms of F-T damage in geopolymers. The factors influencing the F-T resistance of GCs, including raw materials, curing conditions, and modified materials, are meticulously elaborated upon. The results indicate that the F-T resistance of GCs can be significantly enhanced through using high-calcium-content precursors, mixed alkali activators, and rubber aggregates. Moreover, appropriately increasing the curing temperature has been shown to improve the F-T resistance of GCs, especially for those fabricated with low-calcium-content precursors. Among modified materials, the addition of most fibers and nano-materials remarkably improves the F-T resistance of GCs. Conversely, the effect of air-entraining agents on the F-T resistance of GCs seems to be negligible. Furthermore, evaluation and prediction models for the F-T damage of GCs are summarized, including empirical models and machine learning models. In comparison with empirical models, the models established by machine learning algorithms exhibit higher predictive accuracy. This review promotes a more profound understanding of the factors affecting the F-T resistance of GCs and their mechanisms, providing a basis for engineering and academic research.

## 1. Introduction

Ordinary Portland cement (OPC) finds extensive applications in the realm of construction engineering. However, during the manufacturing process of OPC, excessive energy is consumed and the environment is damaged [1,2,3]. Since the production of OPC requires the calcination of limestone, approximately one ton of carbon dioxide is emitted for every ton of cement produced. The CO_2_ emissions from OPC production account for about 8% of the total annual anthropogenic CO_2_ emissions [4,5]. Therefore, it is urgent to develop new environmentally friendly binders to replace cement, which can alleviate serious issues such as environmental pollution [6,7]. Geopolymer, first proposed by Davidovits [8], is a novel aluminosilicate cementitious material that has attracted increasing attention in recent years. Geopolymer composites (GCs) are prepared from precursors such as fly ash (FA), ground granulated blast furnace slag (GGBFS), and metakaolin (MK), along with alkali activators [9]. By replacing cement with geopolymer, industrial by-products can be effectively recycled. Moreover, since calcination is not required in the production of GCs, CO_2_ emissions can be significantly reduced. In addition, GCs possess excellent mechanical properties and chemical resistance. Thus, geopolymer has great potential as a substitute for traditional cementitious composites.

As a potential alternative to OPC, it is necessary to investigate the durability of GCs in complex environments, especially in high-latitude or cold regions, which is of great significance. The freeze–thaw (F-T) cycle gives rise to non-uniform cracks within the concrete matrix. These cracks have a significant detrimental impact on the safety and the service lifespan of concrete structures, potentially leading to structural integrity issues and premature deterioration [10,11]. Several studies have elucidated the F-T damage process of GCs. When subjected to F-T cycling, the free water in the pores of GCs generates repeated crystallization pressure [12] and water pressure [13]. Under the influence of crystallization pressure and water pressure, new microcracks form in the matrix. Such new microcracks allow more water to penetrate into the matrix, ultimately resulting in structural damage to, or even the destruction of, the GC [14,15]. The extent of F-T damage can be quantified using several indices. These include the relative dynamic modulus of elasticity (RDME), weight loss, compressive strength, and ultrasonic pulse velocity (UPV) [9,16,17,18].

The F-T resistance of GCs is significantly affected by a variety of raw materials, which play a crucial role in determining their performance under F-T conditions. Regarding raw materials, the calcium (Ca) content in the precursor is a crucial factor affecting the F-T resistance of GCs. More specifically, when the Ca content in the precursors of GCs is increased, their F-T resistance is significantly strengthened [19]. Additionally, the F-T durability of GCs shows a positive correlation with the increase in the Na/Al ratio, while it decreases as the Si/Al ratio increases. This indicates that a higher Na/Al ratio and a lower Si/Al ratio are beneficial for improving F-T resistance [20]. The properties of alkali activators, including their types, modulus, and dosage, also impact the F-T resistance of GCs [19,21,22,23,24]. For example, Sun et al. [25] found that the RDME loss of GCs activated solely by sodium hydroxide was 32% after 80 F-T cycles, while GCs prepared with a mixed solution of sodium hydroxide and sodium silicate exhibited a significantly lower RDME loss only of 1% after 80 F-T cycles. The properties of the aggregate also influence the F-T resistance of GCs [26,27,28,29,30]. The use of rubber particles (RPs) can significantly decrease the damage brought about by F-T cycles, primarily because of their excellent energy-dissipating ability [28]. Furthermore, the curing temperature is another critical factor affecting the F-T resistance of GCs. Studies have demonstrated that appropriately increasing the curing temperature improves the F-T resistance of these materials [31,32,33]. Research has shown that as the curing temperature increased from 23 °C to 50 °C, the compressive strength loss of a red mud, slurry class F, FA-based geopolymer decreased from 34.4% to 1.63% [31].

To improve the F-T resistance of GCs, many researchers incorporate modified materials such as fibers, nano-materials, and air-entraining agents (AEAs) into GCs [34,35,36,37,38]. Fibers improve the F-T resistance of GCs by restricting the propagation of cracks [39]. One study showed that when compared to a control group that did not contain any steel fibers (SF), GCs that incorporated 0.8% steel fibers (SFs) experienced a reduction in the loss of compressive strength. Specifically, after 200 F-T cycles, this reduction in the loss of compressive strength was roughly 50% [40]. The compressive strength loss in a GC with 0.1% polyethylene (PE) fibers was only 1.4% after 180 F-T cycles, while that of the control group without fibers reached 17.8% [41]. Nano-materials strengthen the F-T durability of GCs by filling matrix pores and facilitating the formation of hydration products [42,43]. For instance, when the graphene oxide (GO) content of a GC was 0.06%, its compressive strength loss was reduced by 41.1% compared to a sample without GO [44]. Ekinci et al. [18] reported that the incorporation of 1% nano-silica (NS) into a GC resulted in a 54% reduction in compressive strength loss after 300 F-T cycles compared to a control group.

**Figure 1:**
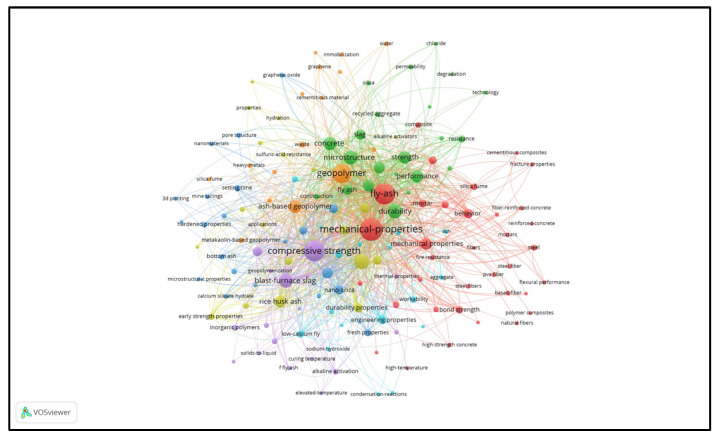
A keyword map of existing reviews’ relevance to GCs, created using VOS viewer 1.6.18.

**Figure 2:**
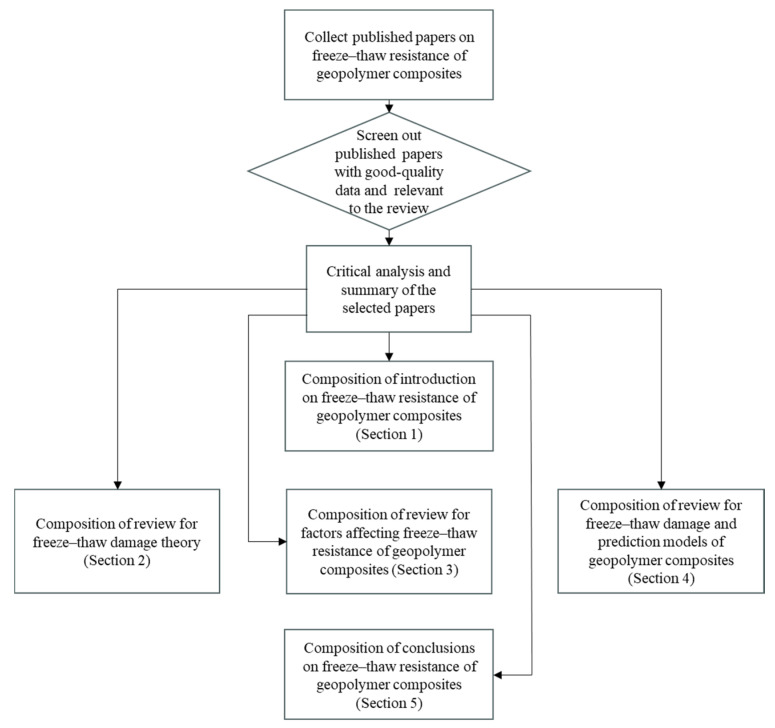
A process diagram for reviewing the F-T resistance of GCs.

Despite numerous reports on the F-T resistance of GCs, the related data is still rather scattered, and comprehensive reviews on this subject are scarce. Figure 1 represents a graphical map of the keywords that are relevant to existing reviews on GCs. The bubble sizes represent the frequency of keywords. From Figure 1, it can be seen that there are almost no existing reviews on the F-T resistance of GCs. Hence, the primary objective of this review is to summarize and conduct a comprehensive review of the relevant literature and data on the F-T resistance of GCs over the past decade. Figure 2 illustrates the process diagram for reviewing the F-T resistance of GCs. The F-T degradation process of GCs and common theories of F-T damage are presented by the review. The impacts of raw materials, curing conditions, and modified materials on the F-T durability of GCs are discussed. Moreover, this review summarizes the evaluation and prediction models for F-T damage of GCs, including empirical models and machine learning (ML) models. This review contributes to a more profound understanding of the factors influencing the F-T resistance of GCs and their underlying mechanisms, thereby providing a foundation for both engineering applications and academic research.

## 2. Freeze–Thaw Damage Mechanism and Theory of Geopolymer Composites

### 2.1. Freeze–Thaw Damage Mechanism of Geopolymer Composites

**Figure 3:**
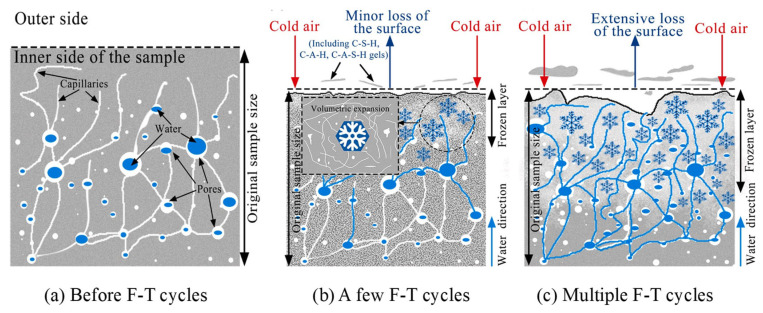
Schematic diagram of F-T damage mechanism of GC [21]. (Reproduced with permission from [21], Elsevier, 2022).

**Figure 4:**
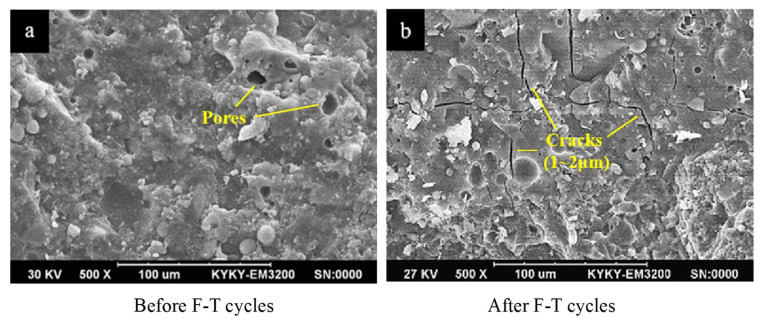
Scanning electron microscope (SEM) images of GC [48]. (Reproduced with permission from [48], Elsevier, 2019).

Figure 3 illustrates the F-T damage process of GCs. The graph reveals that with an increase in the number of F-T cycles, the geopolymer matrix gradually undergoes deterioration. This is manifested as a reduction in the strength, RDME, and weight of the geopolymers [19,40,45]. There are numerous cracks and pores both on the surface and within the geopolymers, as shown in Figure 3a. Before the F-T cycles, water permeates into the cracks and pores of the GC due to the combined effects of capillary pressure and hydraulic pressure. When the temperature decreases, the water in the outer layer of the specimen freezes and forms ice, resulting in the volume expansion of water [46], as depicted in Figure 3b. When the ice crystals grow to fill the entire pore, they exert compressive stress on the pore wall, which is referred to as crystallization pressure. Once the crystallization pressure exceeds the tensile strength of the pore wall, microcracks will initiate in the geopolymer matrix. Additionally, during this process, the unfrozen water is forced into the interior of the matrix, and the migration of water generates a pressure known as hydrostatic pressure [13]. An overly high hydrostatic pressure can also inflict damage on the structure of the geopolymer. After multiple F-T cycles, the internal cracks in the geopolymer interconnect and propagate, forming macroscopic cracks. This leads to cracking of the geopolymer matrix, a reduction in the bonding performance, and surface detachment. After the F-T cycles, numerous cracks develop in the matrix of the GC, significantly compromising the durability and mechanical properties of the material, as illustrated in Figure 4.

The surface peeling of geopolymers is typically a destructive phenomenon under salt-freezing conditions. According to the theory of adhesive peeling [47], when the salt solution on the surface of geopolymer concrete freezes, the ice has a significantly higher expansion coefficient compared to the geopolymer concrete itself. This generates substantial tensile stress at the ice–geopolymer concrete interface, which initiates cracks and causes surface expansion of the geopolymer concrete, ultimately leading to surface detachment. Studies have indicated that salt-freezing inflicts more severe damage on geopolymers compared to water freezing [19]. In summary, whether water or a salt solution is used as the F-T medium, the main reason for the F-T damage of geopolymers is the freezing expansion of the pore solution and the pressure produced during the migration of pore water. The essence of F-T damage in GCs is a process in which the matrix transforms from a dense state to a loose state, accompanied by the initiation, growth, and propagation of microcracks. Macroscopically, this damage is manifested as a significant decrease in mass, RDME, and strength [19,33].

### 2.2. Freeze–Thaw Damage Theory

A great number of studies have investigated the mechanisms of concrete F-T failure, yielding valuable insights. Existing theoretical models of F-T damage can be categorized into two types: one is the icing pressure hypothesis [14], encompassing the crystallization pressure theory, hydrostatic pressure theory, osmotic pressure theory, etc.; the other is the temperature stress hypothesis for high-strength or high-performance concrete [14]. Although these existing mechanisms can, to a certain extent, elucidate certain aspects of the F-T damage phenomena in concrete, there is no single mechanism that can comprehensively explain all the damage phenomena that occur in concrete during the F-T process. Moreover, currently, there is relatively limited research on the F-T damage mechanism of geopolymers, which predominantly relies on the failure mechanism of cement concrete [49]. However, GCs and OPC concrete are both porous materials. As described in Section 2.1, during the F-T cycle, water will migrate and undergo phase transition in the pores of GCs, so some commonly used theories to explain F-T damage in OPC concrete also apply to GCs [50]. Building on previous research achievements, this article expounds on several commonly used theories that are applied to explain the F-T failure of GCs [49,51,52,53,54].

#### 2.2.1. Crystallization Pressure Theory

**Figure 5:**
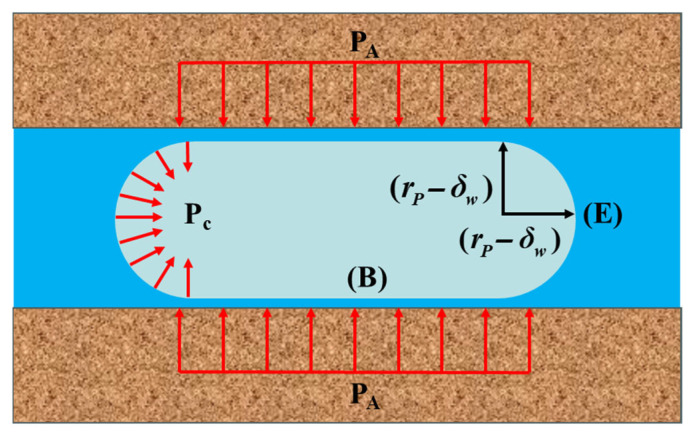
A schematic diagram of a cylindrical ice crystal in a pore of radius *r_p_* [47]. The radii of the cylinder (B) and the hemispherical end (E) are both *r_p_* − *δ_w_*. (Reproduced with permission from [47], Elsevier, 2019).

Figure 5 presents a schematic diagram of cylindrical crystals within the pores of concrete. Due to the uniform temperature around the ice crystal, the ice undergoes simultaneous growth from both ends and the lateral sides, as depicted in Figure 5. However, the lateral growth of the ice crystal is restricted by the pore wall. When the diameter of the ice exceeds the pore size, ice crystals exert a compressive stress P_A_, known as crystallization pressure [12,47]. Cracks form in the pore walls when the crystallization pressure surpasses the tensile strength of the matrix. Once the temperature around the concrete has dropped sufficiently to enable ice crystals to diffuse into most of the pores, the cumulative crystal pressure generated by the ice crystals will lead to the destruction of the concrete structure. The magnitude of the crystallization pressure is influenced by various factors, including the system temperature, the extent of ice formation, the morphology of capillary pores, and the site of initial freezing [47]. The lower the minimum temperature of the F-T cycles, the greater the loss of compressive strength and weight of the GC after F-T cycling [55,56].

#### 2.2.2. Hydraulic Pressure Theory

**Figure 6:**
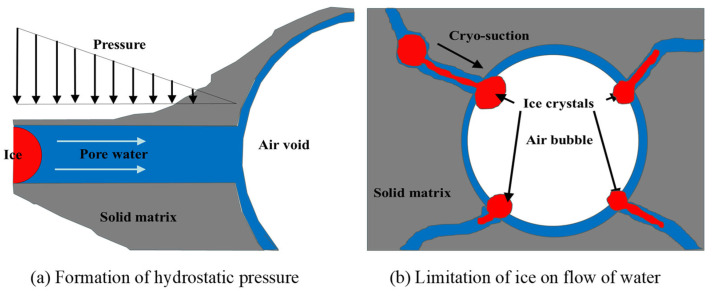
Schematic diagram of hydraulic pressure theory [57]. (Adapted with permission from [57], Elsevier, 2010).

As the temperature decreases to the freezing point, ice forms on the surface of the concrete [57], as depicted in Figure 6a. The volume expansion due to the freezing of pore water causes the unfrozen water to be squeezed into the interior of the concrete, resulting in the formation of hydrostatic pressure [13]. When the hydrostatic pressure exceeds the tensile strength of the matrix, the matrix cracks, generating microcracks. Moreover, ice nucleates and grows at the junction of bubbles and pores, as shown in Figure 6b. This limits the fluidity of the unfrozen water, leading to a further increase in hydrostatic pressure. Under sustained hydrostatic pressure, microcracks within the matrix progressively propagate and coalesce, leading to cumulative damage and material degradation throughout F-T cycles [57]. The formula established by Powers [13] to estimate hydrostatic pressure *P* is presented in Equation (1). According to Equation (1), *P* decreases with a decline in the shortest distance between two bubbles *L*. Therefore, an AEA, which increases the number of bubbles and reduces the pore spacing coefficient, is commonly utilized to boost the F-T resistance of concrete.

(1) $$P=a\prime (1.09-\frac{1}{s})\frac{uR}{k}(\frac{L^{3}}{r_{b}}+\frac{3L^{2}}{2})$$

where *P* represents the hydrostatic pressure, *a′* represents the dynamic viscosity coefficient of water, *s* represents the saturation coefficient of concrete, *u* represents the increase rate of freezing water per 1 °C temperature decrease, *R* represents the cooling rate, *k* represents the coefficient related to concrete permeability, $r_{b}$ represents the average radius of the bubble, and *L* represents the shortest distance between two bubbles.

#### 2.2.3. Osmotic Pressure Theory

**Figure 7:**
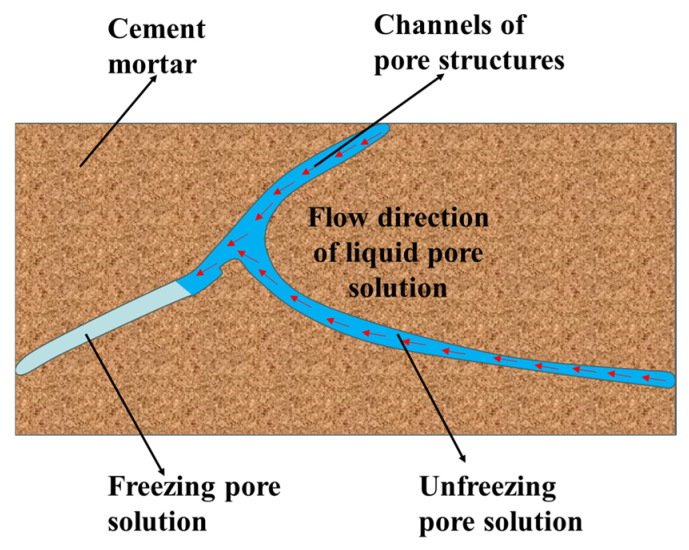
Schematic diagram of osmotic pressure model [60]. (Adapted with permission from [60], Elsevier, 2019).

Concrete, being a porous material, contains numerous pores of different sizes, such as gel pores and bubble pores [58]. When the temperature decreases, a portion of the water in the large pores freezes initially, thereby increasing the salt concentration of the liquid solution inside the large pores. This creates a solution concentration difference between large and small pores. Additionally, the saturated vapor pressure of water is higher than that of ice, causing the unfrozen water to migrate to the frozen areas, as evidenced in Figure 7. Under the combined influence of the solution concentration difference and the saturated vapor pressure difference in the pores, osmotic pressure is generated inside the concrete. When the osmotic pressure surpasses the tensile strength of the concrete, microcracks will initiate and develop within the concrete structure. As the F-T cycle progresses, these microcracks gradually expand, eventually leading to the failure of the concrete structure [59]. Consequently, concrete is prone to damage caused by osmotic pressure in environments with high salt-ion concentrations, such as marine environments. The osmotic pressure theory explains why salt-freezing causes more severe damage to concrete than ordinary freezing. However, the osmotic pressure theory cannot be described by a mathematical model, making it difficult to provide a quantitative explanation of F-T failure.

#### 2.2.4. Glue Spall Theory

**Figure 8:**
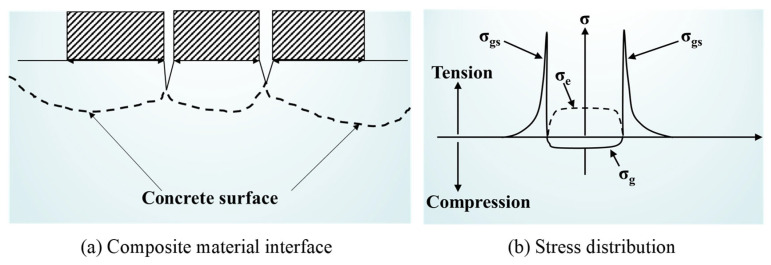
A schematic diagram of the glue spall mechanism [47]. σ_g_, σ_e_, and σ_gs_ are, respectively, the stress generated on the concrete surface, stress in the ice layer, and adhesive peeling stress around the island-shaped ice layer. (Adapted with permission from [47], Elsevier, 2007).

Scherer and Valenza proposed the adhesion peeling theory [47,61,62], which is capable of effectively accounting for the surface scaling phenomenon of concrete following F-T cycles, as depicted in Figure 8. The ice on the surface of concrete forms an ice–concrete interface with the surface of the concrete. Since the shrinkage rate of ice is five times higher than that of concrete, substantial stress is generated at the interface between the ice and the concrete as the temperature drops [63]. With the temperature drops further, the ice layer cracks and forms island-shaped ice layers on the concrete surface. When the island-shaped ice shrinks, stress concentration occurs at the transition region, causing the concrete surface to crack and debond. The magnitude of the stress at the edge of the island-shaped ice layer is related to the initial concentration of the solution. Saltwater ice formed by the freezing of medium-concentration solutions (3%) causes the most severe damage to the concrete surface [61].

## 3. Factors Affecting the Freeze–Thaw Resistance of Geopolymer Composites

### 3.1. Raw Materials

#### 3.1.1. Precursors

Unlike cement composites, GCs are fabricated from various precursors. The F-T resistance of GCs made from different precursors shows significant differences [17,19,64]. Table 1 lists the F-T durability of geopolymers fabricated with different precursors. Evidently, GCs with low-Ca precursors, such as FA and MK, exhibit poor F-T resistance. For example, an FA-based geopolymer concrete was severely damaged after undergoing 21 F-T cycles, losing more than 28% of its mass [19]. Additionally, the results from Liang et al. [65] indicate that the compressive strength loss and weight loss of an MK-based GC were substantial after F-T cycles, reaching 63.30% and 20.24%, respectively. The explanation is that sodium aluminosilicate hydrate (N-A-S-H) gel is the primary hydration product in low-Ca systems [45], containing numerous transition pores that contribute to the poor F-T resistance of low-Ca GCs [48,66]. Moreover, due to the high water absorption of MK, water can easily enter the internal structure of the GC. According to the crystallization pressure theory and osmotic pressure theory, this increases the expansion force of ice during freezing and hydrostatic pressure, resulting in poor F-T resistance of MK-based GCs [67].

Compared to low-Ca GCs, GCs with high-Ca precursors (such as GGBFS) possess superior F-T resistance. Numerous investigations have demonstrated that GGBFS-based geopolymer concrete exhibits satisfactory F-T resistance, with a weight loss of no more than 1.1% after undergoing 300 F-T cycles [17,68]. Furthermore, compared to ordinary Portland cement concrete (OPCC), GGBFS-based geopolymer concrete has better F-T resistance [17]. This can be explained by the fact that calcium aluminosilicate hydrate (C-A-S-H) gel and calcium silicate hydrate (C-S-H) gel, which have a more compact structure compared to N-A-S-H gel, are the major hydration products in GGBFS-based GCs [48,66,69]. Additionally, the fusion of bubbles in the matrix is hindered by slag particles, reducing the average pore size and porosity of GCs [69,70]. These two reasons contribute to the high-Ca GCs having competitive F-T resistance compared to low-Ca GCs. However, Temuujin et al. [71] reported that the F-T durability of a GC prepared with class C FA was lower than that of a GC prepared with class F FA. The reason might be that the crystalline Ca component in class C FA expands when exposed to water, leading to a weakened microstructure and poor F-T resistance.

**Figure 9:**
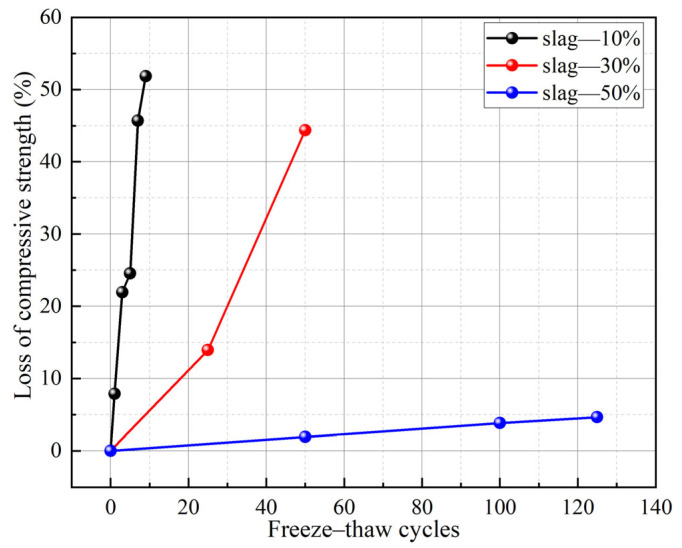
Loss of compressive strength of GCs with different slag contents after F-T cycles [48]. (Adapted with permission from [48], Elsevier, 2019).

**Figure 10:**
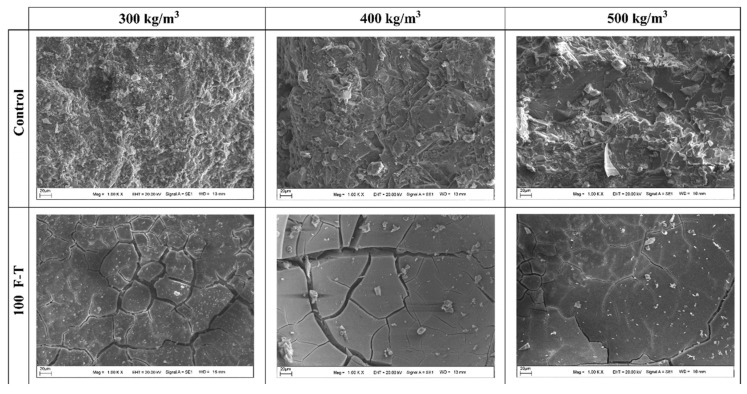
SEM images of GCs with different binder dosages [72]. (Reproduced with permission from [72], Elsevier, 2021).

To strengthen the F-T resistance of low-Ca GCs, part of the low-Ca precursors is replaced with GGBFS to fabricate hybrid-precursor GCs [19,33,48]. Abundant research has shown that the F-T durability of FA-based geopolymer elevates with an increase in the slag proportion in the precursor. For instance, Figure 9 shows that an FA-based geopolymer with 10% slag experienced a 25% loss of compressive strength after just 5 F-T cycles, whereas increasing the slag content to 50% reduced the strength loss to only 4.3% even after 125 F-T cycles [48]. Another study found that the weight loss of a geopolymer with 70% slag was only 0.1% after F-T tests [19]. In addition, the F-T resistance of a GC is also influenced by the dosage of precursor. The geopolymer matrix gradually densifies and the cracks within the matrix decrease with an increase in precursor dosage after F-T cycles, as shown in Figure 10. This leads to a reduction in its permeability and an enhancement in F-T resistance [72]. Moreover, the Na/Al and Si/Al ratios also affect the F-T resistance of MK-based GCs. Research results have indicated that the F-T resistance of an MK-based GC was enhanced with an increase in the Na/Al ratio and decreased with the increase in the Si/Al ratio [20]. This is due to the fact that an elevation in the Na/Al ratio was capable of elevating the compressive strength of the GC.

#### 3.1.2. Activator

**Figure 11:**
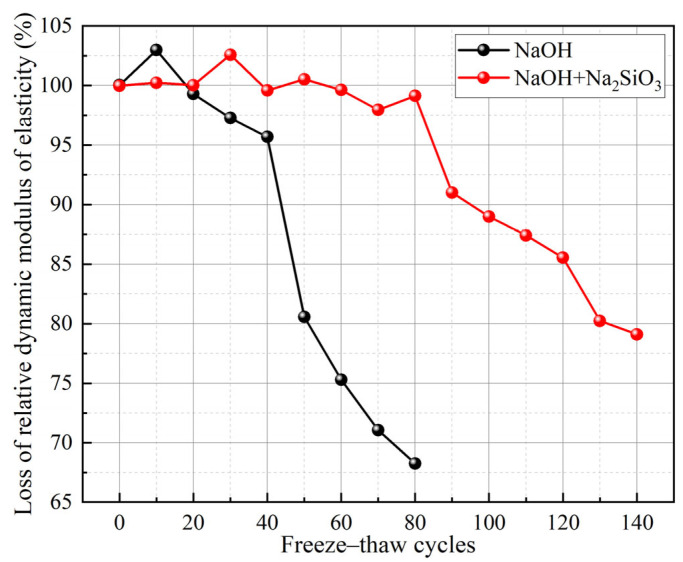
Influence of alkali activator type on F-T resistance of GCs [25]. (Adapted with permission from [25], Elsevier, 2021).

Geopolymers are synthesized through the reaction of precursors with alkali activators. This indicates that the type, modulus, and dosage of alkali activators also influence the F-T resistance of GCs. Min et al. [21] revealed that geopolymers activated solely by sodium hydroxide solution exhibited poor F-T resistance. In contrast, GCs activated with a mixed solution of sodium hydroxide and sodium silicate demonstrated more favorable F-T resistance compared to those activated solely by sodium hydroxide [25], as illustrated in Figure 11. This improvement can be ascribed to the fact that the additional [SiO_4_]^4−^ from sodium silicate enhances the dissolution of Ca^2+^ ions, which promotes the formation of C-A-S-H gel and reduces matrix porosity [21,73]. Furthermore, the F-T resistance of GCs activated by a sodium-based activator is superior to that of GCs activated by a potassium-based activator [22]. This is because the ionic radius of Na⁺ is smaller than that of K⁺. This difference increases the dissolution rate of the precursor, elevates the reaction between the alkaline activator and the silicate precursor, and improves the bonding within the geopolymer structure [22]. A ternary alkali metal activator derived from carbide slag was more effective in improving the F-T resistance of geopolymer concrete based on volcanic ash than a binary alkali metal activator [16]. This is mainly because the ternary alkali activator promoted the dissolution of aluminosilicate precursors and geopolymerization, resulting in an increase in gel production.

**Figure 12:**
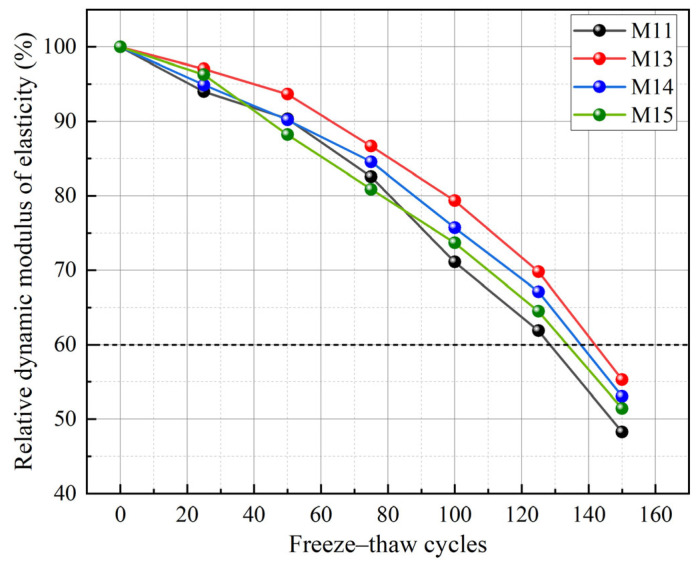
Influence of alkaline activator modulus on RDME [74]. (Adapted with permission from [74], Elsevier, 2023).

Moreover, the modulus of the alkali activator significantly influences the F-T durability of GCs. Zhang et al. [74] reported that the use of an alkaline activator with a modulus of 1.3 (M13) significantly enhanced the freeze–thaw resistance of GCs, as depicted in Figure 12. The reason is that appropriately increasing the modulus of the alkali activator decreases the quantity of harmful pores in the matrix. However, an excessively high modulus of the alkali activator can interfere with the geopolymerization reaction, leading to a decrease in the F-T resistance of GCs. During the curing process, some sodium silicate directly aggregated into solid particles, which reduced the compactness of the GC [67]. In addition, some researchers [24,75] have noted that GCs with higher alkali activator content exhibit better F-T resistance than those with lower activator content. Specifically, after 100 F-T cycles, a GC activated with 20% sodium metasilicate solution exhibited 31% lower compressive strength loss compared to 10% activated counterparts [75]. This is due to the fact that the increase in the content of the alkali activator facilitates the dissolution of the raw materials and the formation of gels, thereby resulting in a more compact matrix [19,23,24]. Nevertheless, Tekle et al. [76] discovered that the RDME loss of a GC with a 0.22 alkali solids-to-binder ratio (AS/B) reached 25%, while the RDME of a GC with a 0.14 AS/B ratio was almost unchanged. This is because an excessive amount of activator solution leads to an excess of water or insufficient alkali reactions in the GC, thereby weakening the adhesive forces within the matrix [77,78]. Overall, GCs activated by a mixture of sodium hydroxide and sodium silicate exhibit better F-T resistance than those activated solely by sodium hydroxide alkaline activator, with the alkaline activator modulus being most effective within the range of 1.3.

#### 3.1.3. Aggregates

**Figure 13:**
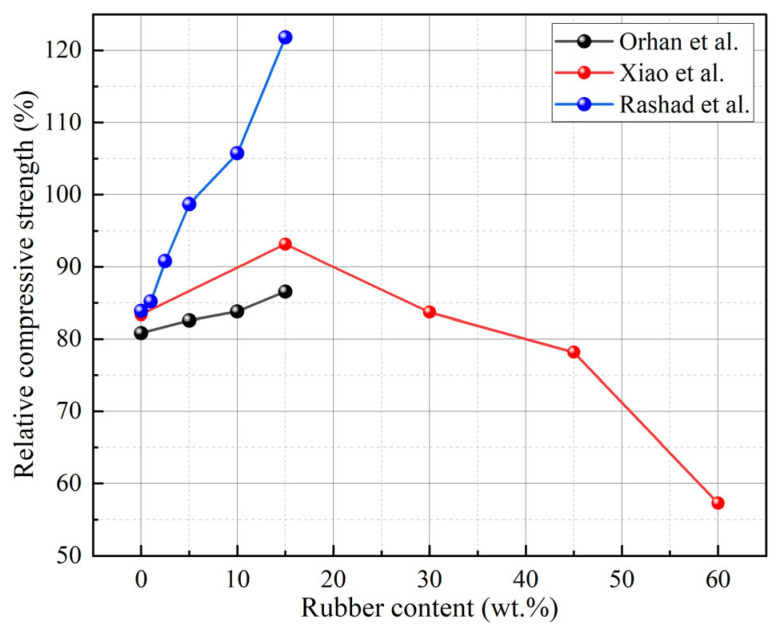
Effect of RPs on relative compressive strength of GCs after F-T cycles [26,27,29]. (Data from [26,27,29]).

**Figure 14:**
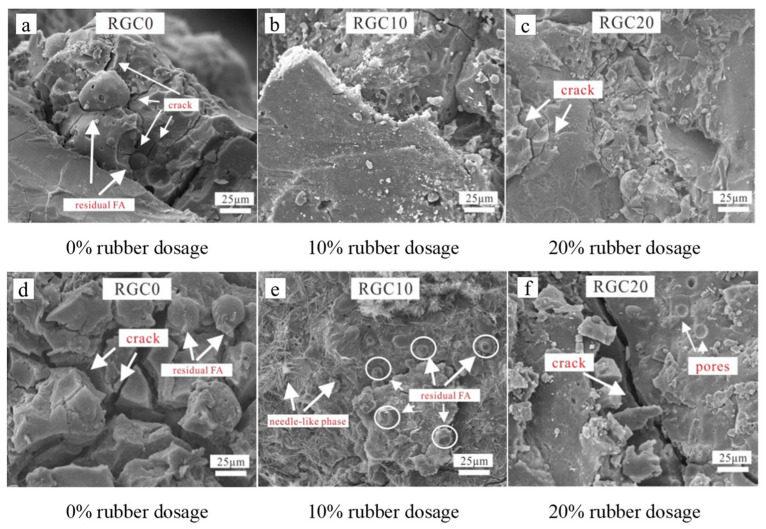
SEM images of rubberized geopolymer concrete before F-T cycles (**a**–**c**); SEM images of rubberized geopolymer concrete after 25 F-T cycles (**d**–**f**) [28]. (Reproduced with permission from [28], Elsevier, 2021).

Aggregates are one of the crucial raw materials for preparing GCs. The F-T durability of GCs is likewise influenced by the types and properties of aggregates. Some industrial wastes, such as RPs and recycled concrete aggregate (RCA), are employed to substitute aggregates during the preparation of GCs [26,27,28,29,30]. Research has revealed that the relative compressive strength of GCs exhibits a pattern of initially rising and subsequently declining as the RP content increases, with an optimal RP content of 15% (Figure 13) [27]. This phenomenon is mainly due to the fact that an excessive addition of RPs will significantly increase the air content within the matrix, leading to the formation of deep and continuous cracks that propagate outward in the matrix. When the mortar on the surface of the concrete peels off, these cracks provide pathways for water to enter the interior of the matrix. Under the action of F-T cycles, these cracks further expand, allowing more water to enter the matrix and exacerbating the damage [79]. When compared with GCs without RPs, those with RPs demonstrated a substantial reduction in cracks following F-T cycles [28], as illustrated in Figure 14. The reason is that RPs absorb a portion of the stress induced by F-T cycles, resulting in a decrease in cracks [80]. Furthermore, the permeability of GCs is decreased by the addition of RPs due to their hydrophobicity. Meanwhile, RPs impede water movement in capillaries, increasing the tortuosity of water transport paths and enhancing the F-T cycle resistance of GCs [81].

**Figure 15:**
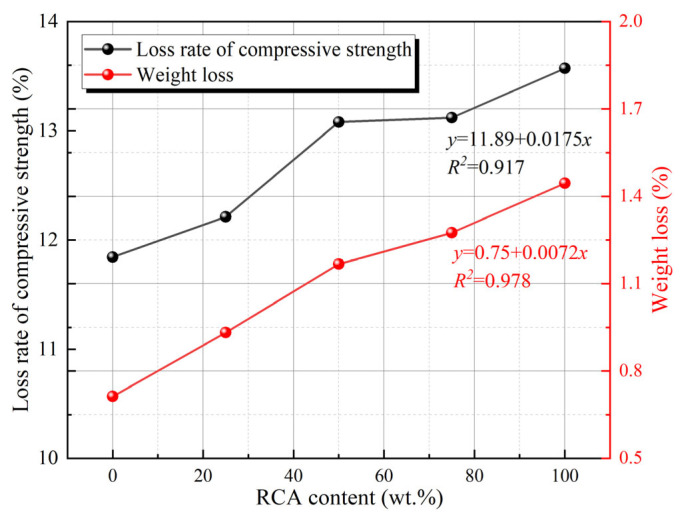
Influence of RCA dosage on the F-T resistance index of geopolymer [72]. (Adapted with permission from [72], Elsevier, 2021).

The utilization of RCA effectively mitigates waste emissions, carbon emissions, and energy consumption. Nevertheless, RCA exhibits higher water absorption and weaker mechanical properties compared to natural aggregates [72]. Consequently, the incorporation of RCA leads to an increase in the amount of water infiltrating into the GC, thereby accelerating the F-T damage of the GC. Although the addition of RCA leads to an elevation in compressive strength loss and weight loss in GCs after F-T cycling, the rate of increase is not substantial, as depicted in Figure 15. Ugurlu et al. [72] reported that the loss of compressive strength of GCs without RCA was 11.8%, whereas that of samples with 100% RCA was 13.5%. This indicates that the utilization of RCA in GCs exerts a minimal impact on their F-T resistance [72,82]. Similarly, using waste glass particles instead of some natural aggregates as aggregates for geopolymer concrete also did not result in a dramatic decrease in the F-T resistance of GCs [83]. In contrast, replacing 100% of natural aggregates with coal gangue resulted in a 70% increase in weight loss after 75 F-T cycles compared to GCs without coal gangue [84]. The cause of this phenomenon is that coal gangue possesses low strength and a loose internal structure. Moreover, the adhesion between coal gangue and the gel material is weak, and there are more voids and pores in the interface transition zone [84].

Apart from the aforementioned aggregates, the utilization of other aggregates can intensify the F-T resistance of GCs. Replacing part of RCA with limestone powder can enhance the F-T resistance of GCs [85]. This is mainly because limestone particles fill the pores among the hydration products, reducing the porosity [86]. Moreover, the nucleation of limestone facilitates the hydration process and the formation of hydration products [87]. Sahin et al. [88] investigated the F-T resistance of GCs prepared with six different types of sand (silica sand, river sand, sandstone, Rilem sand, RCA, and basalt sand (BS)). They discovered that GCs prepared with BS had higher compressive strength and UPV than GCs prepared with several other sands after F-T cycles. This is because the structure of geopolymer mortar filled with BS is more compact and the aggregate paste interface is strengthened. The replacement of natural aggregates with converter steel slag effectively reinforced the F-T durability of geopolymer. This is because the Ca released from the converter steel slag facilitates the formation of C-A-S-H gels. These gels fill the pores and bind the adjacent solid phases together, forming a continuous and dense matrix and a dense interface transition zone [25,89]. In summary, selecting suitable aggregates increases the density of the GC, ameliorates the interface transition zone, and thus enhances the F-T resistance of the GC. Adding 15% RP to GCs is beneficial for improving their F-T resistance, while adding RCA reduces their F-T resistance.

### 3.2. Curing Conditions

In addition to the raw materials used, the curing conditions employed exert a significant influence on the F-T durability of GCs. The suitable curing conditions for GCs synthesized from diverse precursors differ. Table 2 provides a compilation of the impacts that diverse curing conditions have on the F-T resistance of GCs. It can be seen that an appropriate increase in the curing temperature can elevate the F-T resistance of GCs, particularly for low-Ca GCs [65,90,91]. For instance, the F-T durability of a FA–red mud slurry (FA-RMS)-based GC solidified at a temperature of 50 °C and a relative humidity (RH) of 40–50% was determined to be optimal. The reason is that thermal curing accelerates the geopolymerization degree, thereby improving the uniformity and density of the matrix [31,92]. However, thermal curing causes rapid loss of moisture, which increases the cracking tendency in GCs [93]. Therefore, sealed curing is a suggested method, which reduces water evaporation in the structure and avoids cracking of the structure during thermal curing [72,94]. Furthermore, performing dry–wet cycles after thermal curing can reduce the compressive strength loss of geopolymer composite materials by 8.4%, as it promotes the generation of geopolymer gels and strengthens Si-O-Si bonds [95].

The source of Ca in the precursor also affects the suitable curing conditions for a GC. Steam curing effectively improves the F-T resistance of GCs based on class C FA, while GCs that are a mixture of class F FA and slag are more appropriate for standard curing conditions. This is because the steam curing method accelerates the geopolymerization process and densifies the internal structure of class C FA based GCs [33]. However, there are numerous cracks within the GC cured via steam that is based on class F FA mixed with slag. In contrast, under standard curing conditions, the hydration products of the GC based on class F FA mixed with slag are homogeneous and dense, and there is no distinct transition zone between sand and gel [33,98]. Compared with steam-cured GCs, water-cured GCs exhibit lower early-age strength, yet their gel products are more uniformly distributed within the matrix, and the matrix has a lower porosity [96].

### 3.3. Modified Materials

#### 3.3.1. Fiber

**Figure 16:**
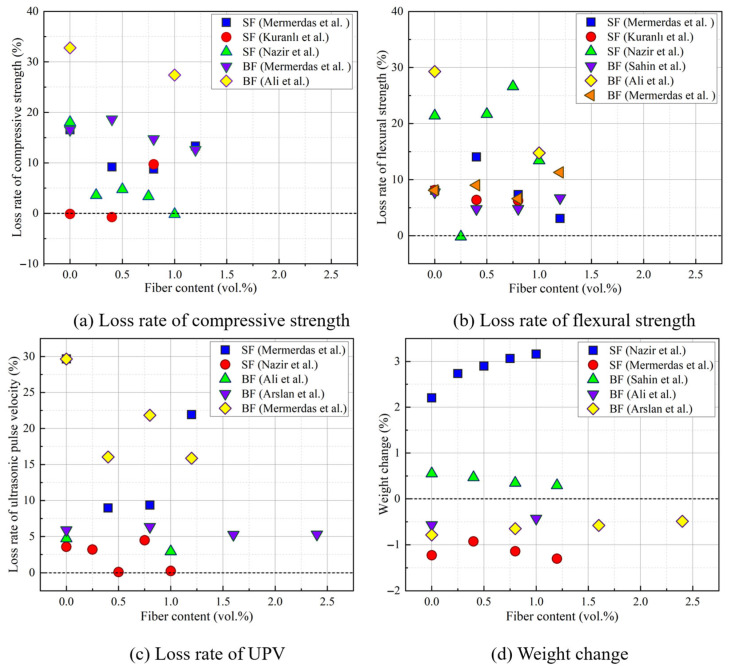
Influence of SF and BF on F-T resistance of GCs [40,41,64,99,100,101,102]. (The date from [40,41,64,99,100,101,102]).

GCs sustain damage during the F-T cycle as a result of the influences of expansion pressure and water pressure. Consequently, some researchers have incorporated fibers, such as SFs and basalt fibers (BFs), into GCs to mitigate F-T degradation. The influence of adding SFs and BFs on the F-T resistance of GCs is illustrated in Figure 16 [40,41,64,99,100,101,102]. Most studies indicate that adding an appropriate amount of SF and BF reduces the loss of mechanical properties in GCs after F-T cycles, such as compressive strength loss, UPV loss, and flexural strength loss [40,41,64]. For example, when compared with control specimens without SF, the compressive strength loss of GCs containing 0.8% SF decreased by approximately 50% (Figure 16a) [40]. The reason is that SFs resist the expansion pressure generated by pore-water freezing through fiber confinement, and also limit the propagation of cracks [39]. In comparison to adding SFs, the effect of adding BFs on the F-T durability of GCs is less significant [40,64]. Nazir et al. [41] observed that the weight of GCs increased, rather than decreased, as shown in Figure 16d. This is because new microcracks inside GCs are generated during F-T cycles. External water infiltrates these microcracks, leading to an increase in weight [41,100].

**Figure 17:**
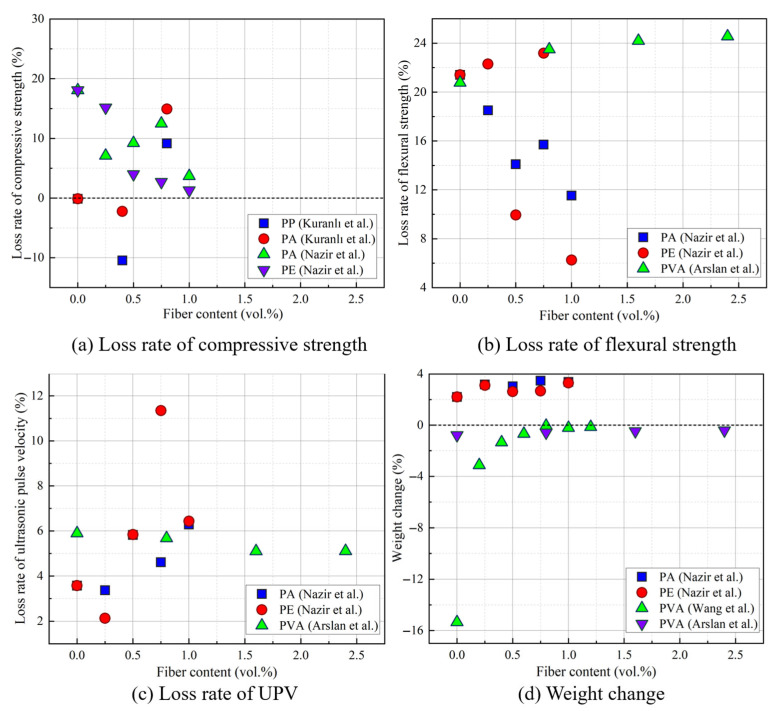
Impact of polymer fibers on F-T resistance of GCs [41,99,101,104]. (Data from [41,99,101,104]).

Adding polymer fibers to GCs can also improve their durability [41,89,101,103]. The effect of polymer fibers on the F-T resistance of GCs is exhibited in Figure 17 [41,99,101,104]. For instance, in contrast to the control group without polypropylene (PP) fibers, the compressive strength loss of GCs with 0.4% PP fibers decreased by approximately 10% after F-T cycles [89]. When compared with the control group without fiber addition, incorporating 1.0% polyethylene (PE) fibers into GCs reduced the loss of compressive strength by approximately 92.70% [41]. Incorporating polyvinyl alcohol (PVA) fibers into GCs effectively reduces weight loss of GCs following F-T cycles [101,104], as depicted in Figure 17d. The reason is that PVA fibers decrease the water absorption rate of GCs. Moreover, PVA fibers act as crack-bridging agents and restrict their propagation, thus reducing the crystallization pressure and water pressure induced by water freezing [89,101,105].

**Figure 18:**
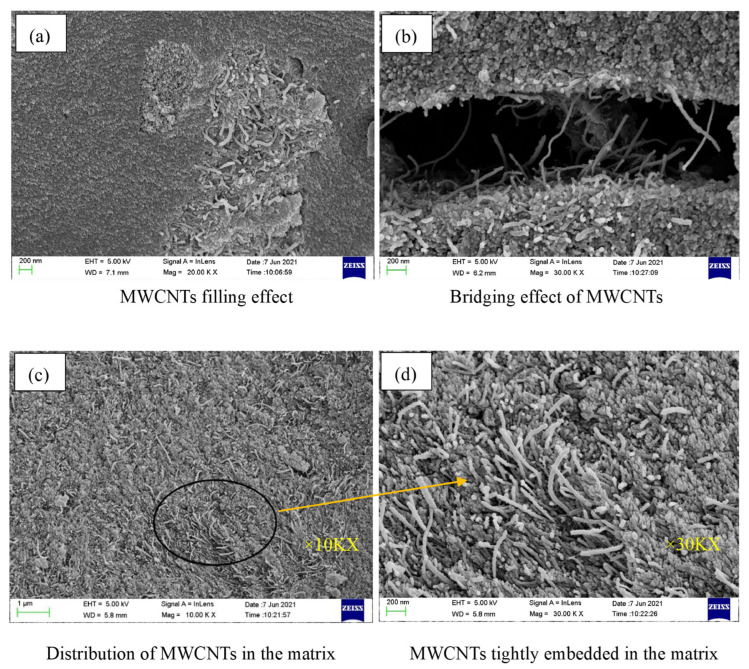
SEM images of GCs with modified MWCNTs incorporated after F-T cycles [14]. (Reproduced with permission from [14], Elsevier, 2022).

Adding 6.0% wood fibers can strengthen the F-T resistance of GCs. This is because wood fibers restrict the propagation of cracks and increase the cohesion of the matrix [106]. The incorporation of 0.1% modified multi-walled carbon nanotubes (MWCNTs) together with 2.0% PVA fibers can augment the number of F-T cycles that GCs can endure [14]. The underlying reason lies in the fact that the crack arrest effect (Figure 18b), mechanical interlocking effect, nano-filling effect (Figure 18a), and nano-nucleation effect of MWCNTs render the geopolymer mortar denser and reduce the capillary porosity of the mortar [14].

#### 3.3.2. Nano-Materials

**Figure 19:**
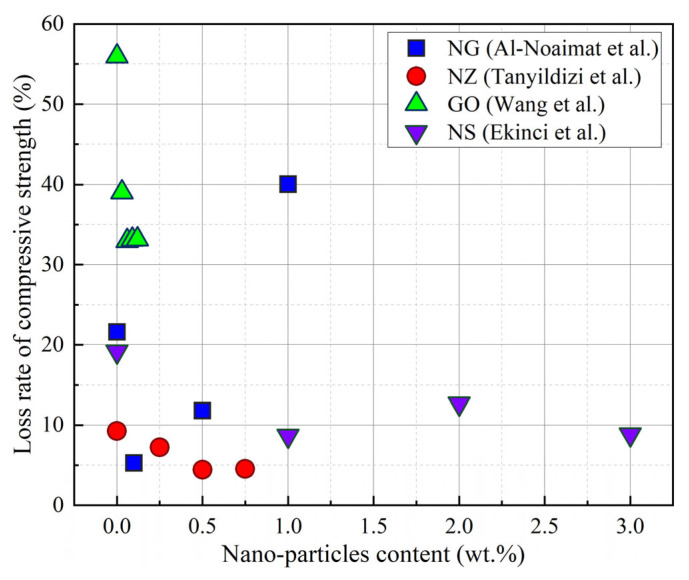
The influence of nano-particles on the loss rate of compressive strength [97,108,109,110]. (Data from [97,108,109,110]).

**Figure 20:**
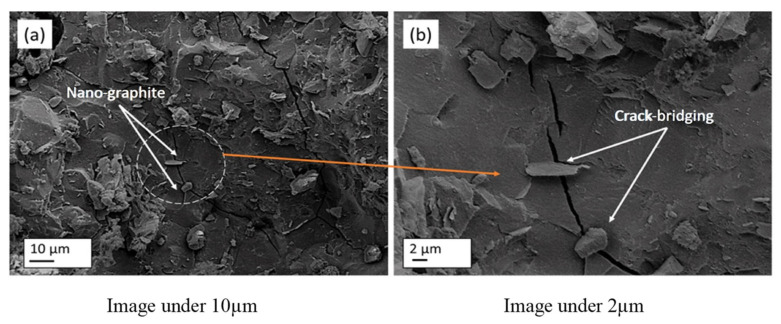
Schematic diagram of NG bridging function [108]. (Reproduced with permission from [108], Elsevier, 2023).

**Figure 21:**
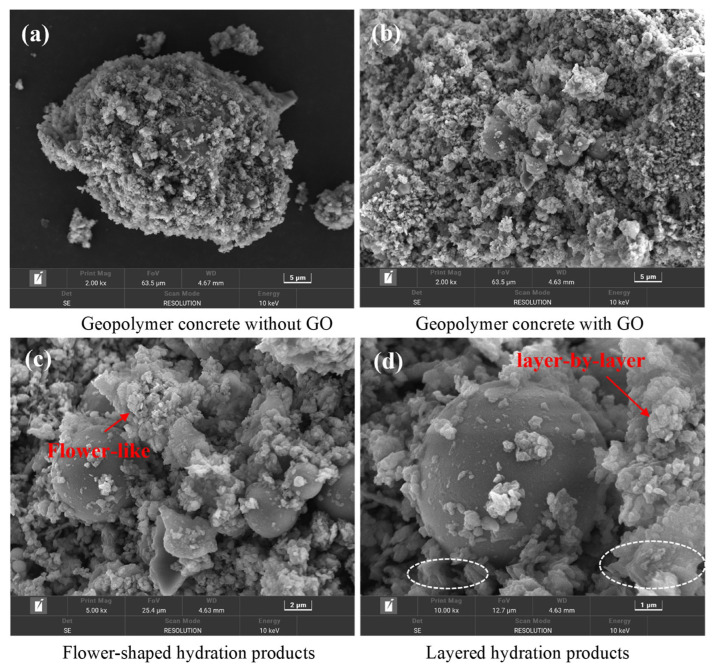
SEM images of geopolymer foam concrete [44]. (Reproduced with permission from [44], Elsevier, 2023).

Due to the fact that nano-materials exhibit excellent volcanic ash and filling effects [107], some researchers have incorporated nano-materials into GCs to elevate their F-T properties. The incorporation of nano-materials into GCs effectively reduces the loss of compressive strength following F-T cycles, as is evident from Figure 19 [97,108,109,110]. For example, when compared with the control group without nano-graphite (NG), a GC containing 0.1% NG exhibited the lowest loss of compressive strength following F-T cycles, with a value amounting to merely 0.5% [108]. This is mainly because NG reduces the water absorption rate of GCs, thus reducing the formation of ice. In addition, during the F-T cycles, NG can also bridge the cracks and limit the propagation of cracks [108], as shown in Figure 20. The incorporation of GO densifies the geopolymer matrix, optimizes the pore structure, and inhibits water transport [109]. Additionally, GO promotes the generation of abundant aluminosilicate gel, such as flower-like and layered hydration products, as presented in Figure 21. When the GO content was 0.06%, the reduction in compressive strength loss reached 41.1% compared to a sample without GO [44].

**Figure 22:**
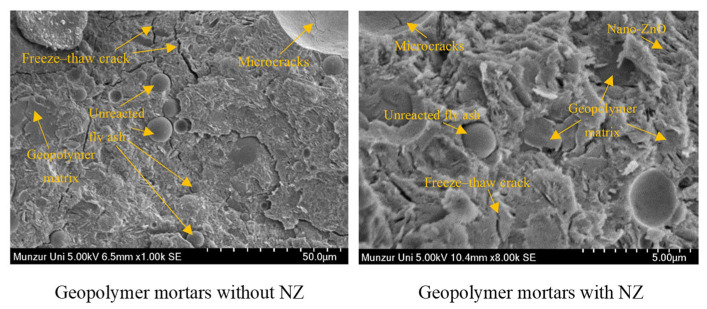
SEM of geopolymer mortars after F-T cycles [111]. (Reproduced with permission from [111], Elsevier, 2024).

NS, nano-alumina (NA), and nano-clay (NC) promote the hydration reaction and the formation of gel products, enhancing the degree of geopolymerization. Moreover, they refine the microstructure of geopolymer and form a dense geopolymer composite slurry owing to their filling effect [34,35,36]. In comparison with NA and NC, NS exerts a more substantial influence on improving the F-T resistance of GCs [97]. The reason for this is that NS provides more silicon dioxide, which increases the generation of geopolymer products [42]. The majority of studies have shown that integrating 1.0% to 2.0% of NS into GCs is capable of improving the F-T resistance of GCs. [18,97,104]. Incorporating nano-zinc oxide (NZ) into geopolymer reduced the loss of compressive strength of GCs following F-T cycles, and the residual compressive strength of a GC containing 0.5% NZ was 95.72% after F-T cycles [110]. The interfacial adhesion between NZ particles and the geopolymer matrix diminishes the range of the interfacial transition zone [110]. Additionally, the existence of nano-zinc oxide (NZ) results in a more compact microstructure of GCs, as shown in Figure 22. However, an excessive incorporation of NZ leads to nano-agglomeration and weakens the F-T resistance of GCs [110]. In conclusion, considering the comprehensive economic benefits of nano-materials and their degree of improvement on F-T resistance, the addition of 1.0% NS is more conducive to enhancing the F-T resistance of GCs than other nano-materials.

#### 3.3.3. Air-Entraining Agents

Numerous studies have demonstrated that AEAs effectively improve the F-T resistance of OPCC [112,113,114,115]. The reason is that AEAs incorporate tiny bubbles (20–50 μm) into concrete, thereby providing space for water expansion and reducing the internal pressure within the concrete [116]. However, the addition of AEAs to GC has not had a significant effect on improving their F-T resistance, as presented in Table 3. Sun et al. [117] discovered that there was no significant difference in the weight loss and RDME loss of FA-based geopolymer mortar with and without an AEA after 300 F-T cycles. The research findings of Aygörmez et al. [90] indicate that an AEA reduced the weight loss of MK-based geopolymer composite materials, but the effect was not substantial. Brooks et al. [118] found that FA-based geopolymers with added AEAs exhibited slight scaling after 40 F-T cycles, whereas FA-based GCs without added AEAs did not show any scaling after 40 F-T cycles. This could potentially be ascribed to the circumstance that the addition of AEA fails to form a homogeneous and stable structure within GCs [118].

## 4. Freeze–Thaw Damage and Prediction Models of Geopolymer Composites

### 4.1. Experience Model

By testing the degradation of GCs under F-T cycling, various F-T resistance indicators can be acquired, which can be quantified to establish a damage model for GCs under F-T cycling. These models assume a pivotal role in assessing the F-T resistance of GCs and forecasting the extent of their degradation. The RDME and compressive strength, which reflect the mechanical properties and internal structure of GCs, are generally used as damage variables to establish F-T damage models [14,84,119]. Common F-T damage models of GCs are summarized in Table 4. Most of the F-T damage models are established using exponential functions or power functions, as listed in Table 4. Compared with those using power functions, models established using exponential functions yield more accurate predictive results. Furthermore, the root mean square error (RMSE) of the nonlinear regression model put forward by Sun et al. [120] for predicting the F-T life of geopolymer concrete was 0.0299, indicating that the model had a satisfactory predictive effect. Porosity is also utilized to develop F-T damage predictive models. Wu et al. [121] developed a prediction model regarding the residual compressive strength of GCs following F-T cycles, as presented in Equations (2) and (3). The coefficient of determination (R^2^) of the model was approximately 0.99, indicating that the model is capable of efficiently predicting the residual compressive strength of geopolymer concrete following F-T cycles.

(2) $$\frac{F_{N}}{F_{0}}=\frac{1-\xi D_{N}}{1-\xi D_{0}}$$

(3) $$D_{i}=1-{\left(1-\frac{\varphi_{i}}{\varphi_{c}}\right)}^{\frac{v}{2}}\cdot {\left(1-\varphi_{i}\right)}^{m+0.5}$$

where *F_N_*, *D_N_*, $\varphi_{i}$ are the compressive strength, degree of damage, and porosity of geopolymer concrete after F-T cycles, respectively; *N* represents the number of F-T cycles; *ξ* is a correction factor; $\varphi_{c}$ is the percolation threshold of the material; ν is the scale index; and m is a parameter related to shrinkage properties.

### 4.2. Machine Learning Models

**Figure 23:**
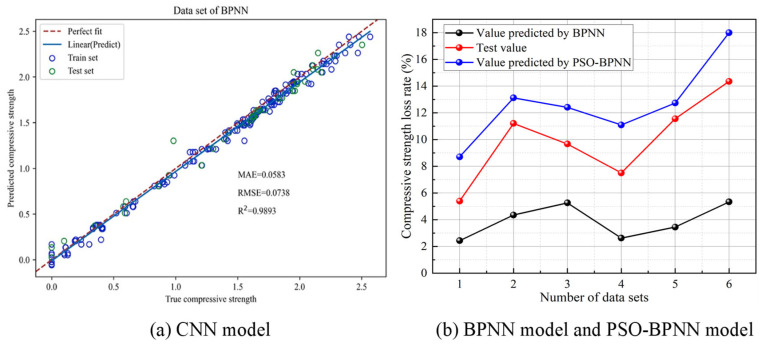
Prediction results of different ML models [124,125]. (Reproduced with permission from [124,125], Elsevier, 2024).

In addition to empirical models, some researchers have also established ML models to predict the F-T damage of GCs. ML is a method of extracting implicit mapping relationships from existing data through model training, aiming to achieve relatively ideal training outcomes and accurate predictions [122]. Deep learning, which is a part of machine learning, makes use of neural networks featuring three or more hidden layers. Through these networks, it is able to learn intricate patterns and acquire meaningful representations. The artificial neural network (ANN) model proposed by Abadel et al. [123] revealed acceptable precision in predicting the F-T resistance of GCs, with an R^2^ value of 0.99. Three ML models, namely backpropagation neural network (BPNN), convolutional neural network (CNN), and gated recurrent unit (GRU), were employed by Yao et al. [124] to forecast the compressive strength of one-part geopolymer after F-T cycles. They used 216 sets of data, with the ratio of the training set to the validation set being 8:2. The input features included the FA/GGBF ratio, freezing temperature, and F-T cycles, while the output variable was compressive strength. Their standard deviations were 0.157, 6.251, 3.917, and 0.716, respectively. The results indicated that all models demonstrated satisfactory predictive performance, suggesting that all three models were reliable in their prediction of the F-T resistance of GCs. Among the three models, the CNN model exhibited the highest prediction accuracy, with an R^2^ value of 0.9966 and an RMSE value of 0.0414, as illustrated in Figure 23a. Moreover, the innovation of metaheuristic algorithms is an approach to optimize neural networks. Algorithms like particle swarm optimization (PSO) and genetic algorithms have the capacity to enhance both the performance and the generalization capability of neural networks. Particle swarm optimization was applied by Zhang et al. [125] to optimize BPNN for the durability prediction of geopolymer mortar. They used 22 sets of data, with 16 sets for training the model and the remaining 6 sets for testing the prediction. The input layer included MK dosage, FA dosage, NS dosage, and PVA fiber dosage, while the output layer was the compressive strength loss rate. Their standard deviations were 3.671, 3.377, 0.807, 0.394, and 3.592, respectively. The predicted results of PSO-BPNN were closer to the actual values than those of BPNN [125], as depicted in Figure 23b. Therefore, it is feasible to predict the F-T durability of GCs accurately and scientifically using PSO-BPNN.

## 5. Conclusions

This review article offers a comprehensive overview of the F-T damage process of GCs, along with some common theories pertaining to F-T damage. It also provides an in-depth and comprehensive summary of the factors that influence the F-T resistance of GCs. By doing so, this review contributes to a more profound understanding of the elements influencing the F-T resistance of GCs and their underlying mechanisms, thereby laying a solid foundation for both engineering applications and academic research. The influencing factors encompass raw materials, curing conditions, and modified materials. Moreover, this review summarizes the models developed through regression analysis and machine learning techniques for the evaluation and prediction of the F-T damage of GCs. The main conclusions of this article are as follows:

(1) The degree of F-T damage of GCs is influenced by pore water pressure and crystallization pressure. The F-T failure process of GCs is predominantly categorized into three distinct stages: water absorption and saturation, ice crystallization during the freezing phase, and microstructural damage leading to macroscopic failure. As the quantity of F-T cycles rises, the surface layer of the geopolymer will start to flake off, and fissures will develop within its internal structure. Three commonly used F-T damage theories are introduced, namely the crystallization pressure theory, hydrostatic pressure theory, and osmotic pressure theory. The theory of crystallization pressure elucidates the process of crystal failure in porous materials.
(2) The Ca content in the precursor can affect the F-T resistance of the geopolymer. GCs with a high Ca content exhibit better F-T resistance than those with a low Ca content. Multiple studies have shown that GCs activated by a mixture of sodium hydroxide and sodium silicate possess better F-T resistance compared to those activated using sodium hydroxide alkaline activator alone. The modulus of the alkaline activator is most effective within the range of 1.3. Replacing natural aggregates with recycled aggregates weakens the F-T resistance of GCs, although this effect is not pronounced. When the proportion of RPs replacing fine aggregates is in the range of approximately 15%, it can augment the F-T resistance of GCs.
(3) The suitable curing conditions for GCs are influenced by the source and amount of Ca contained in the precursor. The F-T resistance of GCs can be enhanced by appropriately increasing the curing temperature to the range of approximately 50 °C to 80 °C, particularly for low-Ca GCs. Desirable results can be obtained when the curing temperature is set within the range of 50 °C to 70 °C.
(4) Fibers, nano-particles, and AEAs are modified materials that influence the F-T resistance of GCs. Extensive experimental studies have demonstrated that fibers can strengthen the F-T resistance of GCs. However, an excessive amount of fibers can form aggregates and cause defects within the matrix. Incorporating an appropriate amount of nano-particles (ranging from 0.45% to 2%) into GCs can improve their F-T resistance. The impact of AEAs on the F-T resistance of GCs is relatively limited, and the findings reported in the literature are still highly controversial.
(5) In this review, the assessment and prediction models of F-T damage were summarized, and the coefficient of determination R^2^ values of these models were all above 0.9. Most of the empirical models for F-T damage were established based on exponential and power functions. When compared with traditional empirical models, most of the prediction models of geopolymer F-T damage established using neural network algorithms possess higher accuracy and broader applicability. Among these models, the prediction results of the CNN model and the ANN model are relatively accurate. Their coefficient of determination R^2^ values are all above 0.99.

## Figures and Tables

**Table 1:** The influence of precursors on the F-T resistance of GCs.

Precursor	Type	Sample Size (mm)	Temperature Range (°C)	F-T Cycles	Compressive Strength Loss	RDME Loss	Weight Loss	Ref.
FA	Geopolymer concrete	100 × 100 cylinder	−22~24	21	/	/	28.20%	[19]
FA/slag	Geopolymer concrete	100 × 100 × 400 prism	−18~4	125	4.30%	53.10%	0.70%	[48]
FA/slag	Geopolymer concrete	100 × 100 cylinder	−22~24	56	/	/	0.10%	[19]
Slag	Geopolymer concrete	100 × 100 × 400 prism	−18~5	300	/	8.51%	−0.48%	[17]
Slag	Geopolymer concrete	100 × 100 × 400 prism	−18~5	300	13.41%	36.33%	1.02%	[68]
FA/slag	Geopolymer mortar	100 × 100 × 400 prism	−18~5	300	/	/	0.80%	[33]
Volcanic tuff	Geopolymer mortar	50 × 50 × 50 cube	−16~3	300	19.70%	37.70%	/	[18]
MK/slag	Geopolymer mortar	50 × 50 × 50 cube	−20~20	90	34.33%	/	0.74%	[64]
MK	Geopolymer mortar	30 × 30 × 30 cube	−18~5	50	63.30%	/	20.24%	[65]

**Table 2:** Impact of solidification conditions on F-T resistance of GCs.

Precursor	Curing Condition	Temperature Range (°C)	Sample Size (mm)	F-T Cycles	Compressive Strength Loss	RDME Loss	Weight Loss	Ref.
FA-RMS	① Sealed curing under 23 °C and RH 40–50%—14 d	−10~4	50.8 × 101.6 cylinder	50	34.40%	/	3.83%	[31]
FA-RMS	① Sealed curing under 23 °C and RH 40–50%—14 d ② Standard curing-14 d	−10~4	50.8 × 101.6 cylinder	44	/	/	20.57%	[31]
FA-RMS	① Sealed curing under 50 °C—7 d ② Sealed curing under 23 °C and RH 40–50%—7 d	−10~4	50.8 × 101.6 cylinder	50	1.63%	/	−3.09%	[31]
FA-RMS	① Sealed curing under 50 °C—7 d ② Sealed curing under 23 °C and RH 40–50%—7 d ③ Ambient curing under 23 °C and RH 40–50%—14 d	−10~4	50.8 × 101.6 cylinder	50	50.2%	/	−9.49%	[31]
FA-RMS	① Sealed curing under 80 °C—24 h ② Sealed curing under 23 °C and RH 40–50%—13 d	−10~4	50.8 × 101.6 cylinder	50	3.83%	/	−8.18%	[31]
FA-RMS	① Sealed curing under 80 °C—24 h ② Sealed curing under 23 °C and RH 40–50%—13 d ③ Ambient curing under 23 °C and RH 40–50%—14 d	−10~4	50.8 × 101.6 cylinder	50	51.21%	/	−2.87%	[31]
MK	① Sealed curing under 60 °C—3 d ② Sealed curing—25 d	−20~20	100 × 100 × 100 cube	90	34.28%	/	0.74%	[64]
MK	① Sealed curing under 60 °C—3 d ② Sealed curing—25 d	−18~4	100 × 100 × 400 prism	300	28.70%	/	0.43%	[90]
MK	① Steam curing under 50 °C—28 d	−18~5	30 × 30 × 30 cube	50	63.30%	/	20.24%	[65]
Class F FA–Slag	① Standard curing—24 d	−18~5	100 × 100 × 400 prism	100	/	−2.41%	0.12%	[33]
Class F FA–Slag	① Steam curing under 60 °C—24 h ② Standard curing—23 d	−18~5	100 × 100 × 400 prism	100	/	40.12%	−0.47%	[33]
Class C FA	① Standard curing—24 d	−18~5	100 × 100 × 400 prism	100	/	3.89%	16.50%	[33]
Class C FA	① Steam curing under 60 °C—24 h ② Standard curing—23 d	−18~5	100 × 100 × 400 prism	100	/	4.51%	1.05%	[33]
Slag	① Steam curing under 85 °C—12 h ② Standard curing—27.5 d	−20~20	40 × 40 × 160 prism	150	17.05%	/	1.77%	[96]
Slag	① Standard curing—24 d	−18~5	100 × 100 × 400 prism	150	21.87%	6.74%	0.54%	[68]
Slag	① Water curing—90 d	−18~4	100 × 100 × 100 cube	300	6.14%	/	4.50%	[97]
Slag	① Sealed curing under 80 °C—24 h ② Water curing—27 d	−18~4	50 × 50 × 50 cube	100	12.04%	/	0.71%	[72]

Note: ①, ②, and ③ refer to the curing sequence.

**Table 3:** Influence of AEAs on F-T resistance of GCs.

Type	AEA Content	Sample Size (mm)	Temperature Range (°C)	F-T Cycles	Compressive Strength Loss	RDME Loss	Weight Loss	Ref.
Geopolymer mortar	0%	23.5 × 23.5 × 50.8 prism	−18~4	300	5.00%	8.40%	0.12%	[117]
Geopolymer mortar	0.20%	23.5 × 23.5 × 50.8 prism	−18~4	300	0%	6.80%	0.17%	[117]
Geopolymer mortar	0%	100 × 100 × 400 prism	−18~4	300	28.31%	/	0.43%	[90]
Geopolymer mortar	0.10%	100 × 100 × 400 prism	−18~4	300	23.73%	/	0.12%	[90]

**Table 4:** Empirical model of F-T damage.

F-T Evaluation Model	F-T Damage Model	Evaluating Index	Ref.
$w=1-ae^{bN}$	$w=1-E_{N}/E_{0}$	R^2^: 0.910~0.986	[14]
$w=aN^{b}$	$w=1-E_{N}/E_{0}$	R^2^: 0.936~0.992	[84]
$w=a(1-e^{-bN})$	$w=1-E_{N}/E_{0}$	R^2^: 0.974~0.996	[84]
$w=a\cdot e^{-bN}$	$w=F_{N}/F_{0}$	R^2^: 0.950	[15]
$w=a\cdot N^{b}$	$w=F_{N}/F_{0}$	R^2^: 0.790	[15]
$w=a\cdot e^{-bN}$	$w=1-F_{N}/F_{0}$	R^2^: 0.958	[15]
$w=a\cdot N^{b}$	$w=1-F_{N}/F_{0}$	R^2^: 0.998	[15]
$w=a\cdot e^{-bN}$	$w=E_{N}/E_{0}$	R^2^: 0.860	[15]
$w=aN^{b}$	$w=E_{N}/E_{0}$	R^2^: 0.600	[15]
$w=a\cdot e^{-bN}$	$w=1-E_{N}/E_{0}$	R^2^: 0.971	[15]
$w=aN^{b}$	$w=1-E_{N}/E_{0}$	R^2^: 0.897	[15]
$w=a\cdot e^{bN}$	$w=1\pm \frac{E_{N}/E_{0}-0.60}{0.40}\cdot \frac{0.25-F_{N}/F_{0}}{0.25}$	RSME: 0.030	[120]

Note: *w* is the F-T damage index, *E_N_* is the RDME under *N* F-T cycles, *E*_0_ is the RDME before the F-T cycles, and *a* and *b* are constant.

## Data Availability

No new data were created or analyzed in this study.

## Abbreviations

OPC	ordinary Portland cement
GC	geopolymer composite
FA	fly ash
GGBFS	ground granulated blast furnace slag
MK	metakaolin
F-T	freeze–thaw
RDME	relative dynamic modulus of elasticity
UPV	ultrasonic pulse velocity
Ca	calcium
AEA	air-entraining agent
SF	steel fiber
PE	polyethylene
GO	graphene oxide
RP	rubber particle
NS	nano-silica
ML	machine learning
SEM	scanning electron microscopy
N-A-S-H	sodium aluminosilicate hydrate
OPCC	ordinary Portland cement concrete
C-S-H	calcium silicate hydrate
C-A-S-H	calcium aluminosilicate hydrate
RCA	recycled concrete aggregate
BS	basalt sand
FA-RMS	fly ash–red mud slurry
RH	relative humidity
BF	basalt fiber
PP	polypropylene
PVA	polyvinyl alcohol
MWCNTs	multi-walled carbon nanotubes
NG	nano-graphite
NA	nano-alumina
NC	nano-clay
NZ	nano-zinc oxide
RMSE	root mean square error
ANN	artificial neural network
BPNN	backpropagation neural network
CNN	convolutional neural network
GRU	gated recurrent unit
PSO	particle swarm optimization
